# HALP (Hemoglobin, Albumin, Lymphocyte, and Platelet) Score and Its Companions in Papillary Thyroid Cancer: Can They Predict Central Lymph Node Metastasis?

**DOI:** 10.7759/cureus.76801

**Published:** 2025-01-02

**Authors:** Mahmut Kaan Demircioğlu, Serkan Sari, Mehmet Saban Korkmaz, Gorkem Yildiz, Sezer Akbulut, Tugba Matlim Ozel, Yigit Soytas

**Affiliations:** 1 Surgical Oncology, Basaksehir Cam and Sakura City Hospital, Istanbul, TUR; 2 General Surgery, Basaksehir Cam and Sakura City Hospital, Istanbul, TUR

**Keywords:** halp score, lymph node metastasis, papillary thyroid cancer, predictive factor, prognostic nutritional index (pni)

## Abstract

Background

Papillary thyroid cancer (PTC) is one of the most common cancers with a favorable prognosis. The prognosis is most commonly associated with lymph node metastasis. We investigated whether there is a relationship between the hemoglobin, albumin, lymphocyte, and platelet (HALP) score and PTC central region lymph node metastasis in the preoperative period. NLR (neutrophil/lymphocyte ratio), PLR (platelet/lymphocyte ratio), MLR (monocyte/lymphocyte ratio), PIV (pan-immune-inflammation value), PNI (prognostic nutritional index), SII (systemic immune-inflammation index) were analyzed as other predictive factors for PTC central region lymph node metastasis.

Methods

The data of 235 patients who were operated on for PTC and underwent additional central neck dissection in our clinic were analyzed retrospectively. The effect of the HALP score, NLR, PLR, MLR, PIV, PNI, and SII values on PTC central region lymph node metastasis was analyzed with univariate and multiple logistic regression analyses.

Results

The HALP score, NLR, PLR, MLR, PIV, PNI, and SII had no statistically significant effect on the number of positive lymph nodes in the central region, lymphatic invasion, vascular invasion, extra-thyroidal invasion in PTC (p>0.050 for each). For PTC, stage progression was associated with a decrease in preoperative PNI (OR=0.871; p=0.019).

Conclusion

There was a significant negative correlation between PNI and the stage of the PTC. No significant correlation was found between the HALP score and prognostic predictors of papillary PTC central lymph node metastasis. This may be related to the fact that PTC is a type of cancer that does not affect biochemical parameters much.

## Introduction

The hemoglobin, albumin, lymphocyte, and platelet (HALP) score is a prognostic immunonutritional biomarker that has recently been used in malignancy patients as well as in many other diseases [1,2]. It has been reported to have a negative prognostic effect on many different organ malignancies.

According to the updated tumor, node, metastasis (TNM) staging of the American Joint Committee on Cancer (AJCC), it has been emphasized that lymph node status at the time of diagnosis and distant metastasis are the most important prognostic factors in differentiated thyroid cancers [3].

Although definitive surgical treatment is performed in many patients with papillary thyroid cancer (PTC), lymph node metastasis occurs in the central or lateral neck compartments during follow-up. This raises the question of whether there is a lymph node that was not detected or was missed in preoperative imaging and intraoperative imaging [4,5].

We investigated whether it is possible to predict the risk of lymph node metastasis, which has not yet been detected by preoperative imaging methods, with a non-invasive method such as the HALP score, NLR (neutrophil/lymphocyte ratio), PLR (platelet/lymphocyte ratio), MLR (monocyte/lymphocyte ratio), PIV (pan-immune-inflammation value), PNI (Prognostic Nutritional Index), or SII (Systemic immune-inflammation index), which can be easily calculated. We investigated the prognostic value of the HALP score and the others in terms of disease severity and central lymph node metastasis.

## Materials and methods

Prior to the study, ethics approval was obtained from the local ethics committee of Basaksehir Cam and Sakura City Hospital with approval number 2023-344. The study was designed in accordance with the Declaration of Helsinki.

The data of the patients operated for PTC between 2020 and 2023 in our clinic were retrospectively analyzed. A total of 235 patients over 18 years of age, regardless of gender, who were diagnosed with PTC by thyroid fine-needle aspiration biopsy performed during preoperative examinations and underwent prophylactic or therapeutic central neck dissection, were included in the study.

Patients with benign fine-needle aspiration biopsy results or patients who underwent surgery for benign reasons and did not receive neck dissection because malignancy was detected in the final pathology report and patients with hematological diseases were excluded from the study. After excluding patients with comorbidities or additional malignancies that would affect immunonutritional status, the data of a total of 200 patients who were eligible for the study were analyzed.

HALP score calculated as = [hemoglobin (g/L) × albumin (g/L) × lymphocyte (/L)] / platelet (/L) [2].

NLR was calculated as neutrophil count (103/mm^3^) /lymphocyte count(103/mm^3^), PLR was calculated as platelet count (103 uL)/lymphocyte count (103/mm^3^), MLR was calculated as monocyte count (103/mm^3^)/lymphocyte count (103/mm^3^).

PIV was calculated as (neutrophil count (103/mm^3^) × platelet count (103 uL) × monocyte count (103/mm^3^))/lymphocyte count (103/mm^3^). SII was calculated as (neutrophil count × platelet count)/lymphocyte count.

PNI was calculated as 10 × serum albumin level (g/dL) + 0.005 × total lymphocyte count (per mm^3^).

SII was calculated as neutrophil count (103/mm^3^) × platelet count (103 uL)/lymphocyte count (103/mm^3^) [6].

Statistical analysis

Data were analyzed with IBM SPSS V23. Binary logistic regression analysis was used to analyze the risk factors affecting the presence of metastatic central lymph node, lymphatic invasion, vascular invasion, extra-thyroidal invasion, and stage (according to TNM 8th edition). Linear regression analysis was used to analyze the independent variables affecting tumor diameter and HALP score. ROC analysis was used to determine the cut-off values of the parameters. The statistical significance level was taken as p<0.050.

## Results

Of the 200 patients who were eligible for inclusion in the study, 162 (81%) were female and 38 (19%) were male. The mean age of the patients was 43.56 years.

The effects of the HALP score and the NLR, PLR, MLR, PIV, PNI, and SII indices on the presence of metastatic lymph nodes, lymphatic invasion, vascular invasion, extra-thyroidal invasion, and TNM stage were analyzed by univariate and multiple logistic regression analysis. In the analyses performed according to the presence of metastatic lymph nodes, variables such as the HALP score, NLR, PLR, MLR, PIV, PNI, and SII were evaluated as independent variables, and their relationships with the presence of metastatic lymph nodes were examined. 

In these analyses, none of the variables had a statistically significant effect on the presence of metastatic lymph nodes (p>0.050). Similarly, the effect of variables such as the HALP score, NLR, PLR, MLR, PIV, PNI, and SII were evaluated in analyses related to lymphatic invasion, vascular invasion, and extra-thyroidal spread. However, no statistically significant effect of the variables was observed in these analyses (p>0.050).

In the univariate model, a statistically significant effect of PNI score on the TNM stage was found and a protective effect of increasing PNI score on the TNM stage was obtained (OR=0.871; p=0.019). PNI was found to be higher at lower stages and was found to be statistically significant. The effect of other variables was not significant (p>0.050) (Table 1). 

**Table 1:** The effect of the HALP score and NLR, PLR, MLR, PIV, PNI, and SII indices on metastatic lymph node, lymphatic invasion, vascular invasion, extra-thyroidal spread, and TNM stage HALP: hemoglobin, albumin, lymphocyte, and platelet score, NLR: neutrophil/lymphocyte ratio, PLR: platelet/lymphocyte ratio, MLR: monocyte/lymphocyte ratio, PIV: pan-immune-inflammation value, PNI: prognostic nutritional index, SII: systemic immune-inflammation index; TNM: tumor, node, metastasis

Dependent variable	Independent variable	Univariate		Multiple	
		OR (%95 CI)	p	OR (%95 CI)	
Metastatic lymph node	HALP	0.938 (0.858 – 1.026)	0.161	0.93 (0.824 - 1.05)	0.244
	NLR	1.232 (0.911 – 1.666)	0.175	1.188 (0.743 - 1.899)	0.472
	PLR	1.004 (0.997 – 1.01)	0.255	0.999 (0.99 - 1.008)	0.783
	MLR	3.117 (0.044 – 222.102)	0.601	0.096 (0 - 34.138)	0.434
	PIV	1.001 (0.999 - 1.003)	0.239	1.001 (0.998 - 1.003)	0.662
	PNI	1.031 (0.951 - 1.119)	0.456	1.032 (0.947 - 1.125)	0.470
	SII	1.001 (1 - 1.002)	0.124	---	---
Lymphatic invasion	HALP	1.025 (0.917 - 1.146)	0.664	1.186 (0.869 - 1.617)	0.283
	NLR	1.192 (0.823 - 1.726)	0.353	1.13 (0.619 - 2.065)	0.691
	PLR	1.004 (0.997 - 1.012)	0.262	1.012 (0.995 - 1.029)	0.185
	MLR	0.789 (0.005 - 117.684)	0.926	0.076 (0 - 87.709)	0.473
	PIV	1.001 (0.999 - 1.003)	0.436	1 (0.997 - 1.003)	0.873
	PNI	1.037 (0.941 - 1.142)	0.463	0.991 (0.885 - 1.109)	0.869
	SII	1.001 (1 - 1.002)	0.194	---	---
Vascular invasion	HALP	1.004 (0.899 - 1.121)	0.947	0.951 (0.759 - 1.192)	0.666
	NLR	1.184 (0.885 - 1.585)	0.254	1.19 (0.73 - 1.938)	0.486
	PLR	0.997 (0.988 - 1.006)	0.480	0.988 (0.973 - 1.004)	0.134
	MLR	14.398 (0.054 - 3829.327)	0.349	4.358 (0.002 - 9809.138)	0.709
	PIV	1.001 (0.999 - 1.003)	0.190	1.001 (0.998 - 1.005)	0.423
	PNI	0.991 (0.887 - 1.108)	0.876	1.012 (0.899 - 1.14)	0.838
	SII	1 (0.999 - 1.001)	0.382	---	---
Extra-thyroidal spread	HALP	1.048 (0.936 - 1.172)	0.415	1.049 (0.918 - 1.199)	0.484
	NLR	0.728 (0.353 - 1.5)	0.389	0.475 (0.131 - 1.714)	0.255
	PLR	0.996 (0.984 - 1.008)	0.505	1.001 (0.986 - 1.017)	0.871
	MLR	0.232 (0 - 916.135)	0.729	1.171 (0 - 121619.431)	0.979
	PIV	1 (0.997 - 1.003)	0.883	1.003 (0.998 - 1.008)	0.219
	PNI	0.916 (0.785 - 1.069)	0.264	0.923 (0.781 - 1.091)	0.348
	SII	1 (0.998 - 1.001)	0.725	---	---
TNM stage	HALP	0.98 (0.862 - 1.114)	0.755	0.951 (0.759 - 1.193)	0.666
	NLR	0.852 (0.554 - 1.31)	0.465	1.199 (0.637 - 2.256)	0.574
	PLR	0.995 (0.986 - 1.004)	0.263	0.994 (0.979 - 1.01)	0.457
	MLR	1.95 (0.006 - 608.106)	0.820	55.372 (0.015 - 200363.072)	0.337
	PIV	0.998 (0.996 - 1.001)	0.192	0.997 (0.993 - 1.001)	0.143
	PNI	0.871 (0.775 - 0.978)	0.019	0.889 (0.783 - 1.01)	0.071
	SII	0.999 (0.998 - 1.001)	0.224	---	---

The linear regression model established to examine the effect of the HALP score and NLR, PLR, MLR, PIV, PNI, and SII indices on tumor diameter was not statistically significant (F=1.26; p=0.278) (Table 2).

**Table 2:** The effect of the HALP score and NLR, PLR, MLR, PIV, PNI, and SII indices on tumor diameter F=1.26, p=0.278, R2=0.038, adjusted R2=0.008, β1: unstandardized beta coefficient, β2: standardized beta coefficient, r1: zero-order correlation, r2: partial correlation, VIF: variance inflation factor, SE: standard error, HALP: hemoglobin, albumin, lymphocyte, and platelet score, NLR: neutrophil/lymphocyte ratio, PLR: platelet/lymphocyte ratio, MLR: monocyte/lymphocyte ratio, PIV: pan-immune-inflammation value, PNI: prognostic nutritional index, SII: systemic immune-inflammation index

	β1 (%95 CI)	SE	β2	t	p	r1	r2	VIF
Constant	18.589 (-0.74 - 37.919)	9.8		1.897	0.059			
HALP	0.122 (-0.388 - 0.631)	0.258	0.042	0.471	0.638	0.023	0.034	1.567
NLR	-0.073 (-2.033 - 1.886)	0.994	-0.008	-0.074	0.941	0.042	-0.005	2.456
PLR	-0.026 (-0.068 - 0.015)	0.021	-0.126	-1.251	0.213	-0.052	-0.09	2.042
MLR	23.661 (-4.607 - 51.929)	14.332	0.158	1.651	0.1	0.119	0.118	1.832
PIV	0.004 (-0.008 - 0.016)	0.006	0.072	0.654	0.514	0.084	0.047	2.409
PNI	-0.191 (-0.607 - 0.225)	0.211	-0.067	-0.906	0.366	-0.086	-0.065	1.086

**Figure 1:**
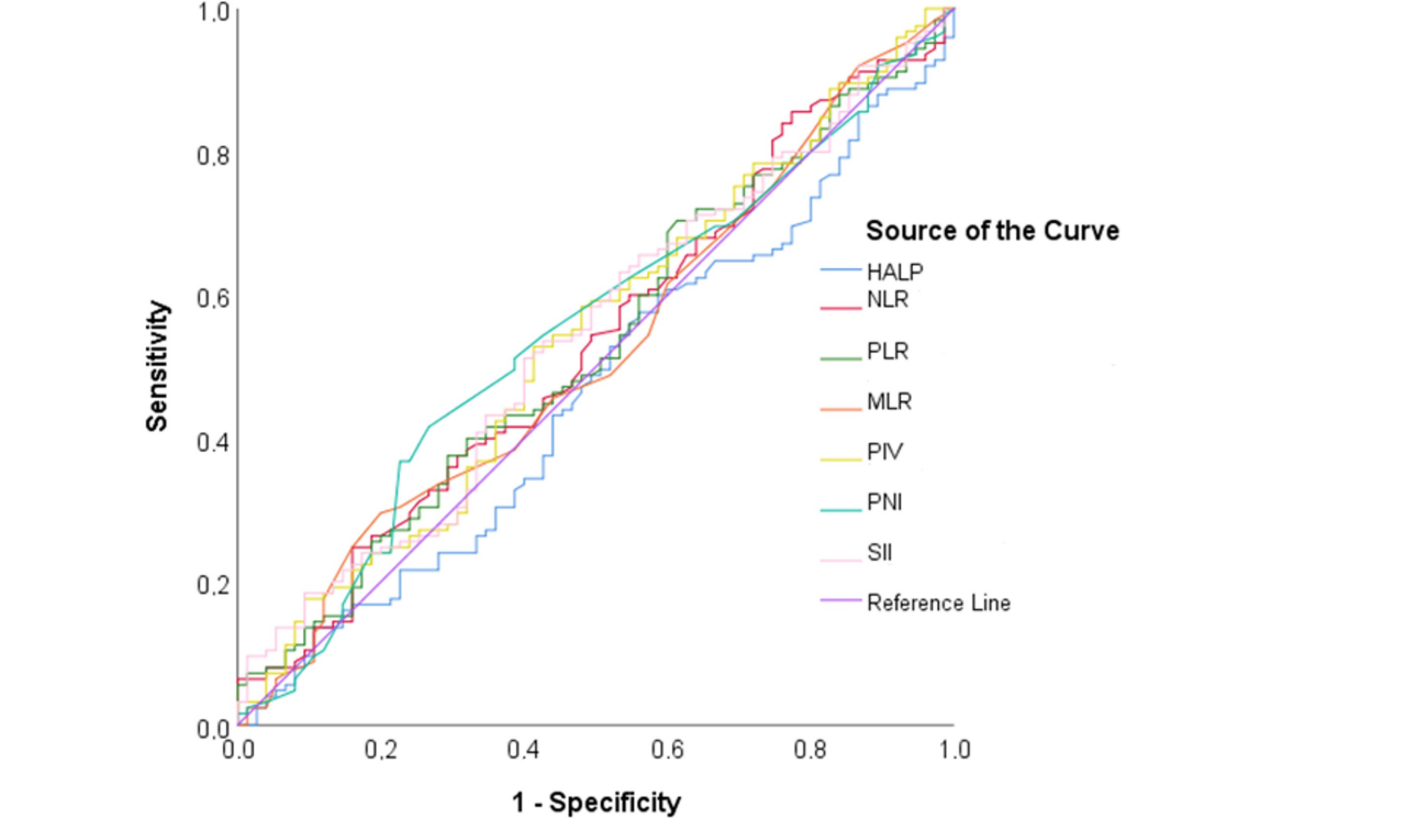
ROC curve of parameters for predicting the presence of a metastatic central lymph node ROC: receiver operating characteristic

As a result of ROC analysis, all AUC values of the parameters were not statistically significant (p>0.050) (Figure 1) (Table 3).

**Table 3:** ROC analysis of parameters for predicting the presence of a metastatic central lymph node ROC: receiver operating characteristic, HALP: hemoglobin, albumin, lymphocyte, and platelet score, NLR: neutrophil/lymphocyte ratio, PLR: platelet/lymphocyte ratio, MLR: monocyte/lymphocyte ratio, PIV: pan-immune-inflammation value, PNI: prognostic nutritional index, SII: systemic immune-inflammation index

	AUC (%95 CI)	p
HALP	0.465 (0.383 - 0.547)	0.409
NLR	0.532 (0.45 - 0.614)	0.449
PLR	0.529 (0.447 - 0.611)	0.489
MLR	0.521 (0.438 - 0.604)	0.618
PIV	0.54 (0.457 - 0.623)	0.343
PNI	0.547 (0.465 - 0.629)	0.266
SII	0.544 (0.462 - 0.625)	0.303

## Discussion

Although the incidence of PTC is increasing, it is a malignancy with a very good prognosis. The most important factor for prognosis, recurrence, and reoperations is cervical lymph node metastasis [7]. Methods that can predict lymph node metastasis with preoperative non-invasive tests are being investigated.

In our study, it was observed that the stage decreased with increasing PNI in terms of PTC central zone metastasis. PNI is a nutritional prognostic factor that can and should be easily calculated preoperatively in malignancy patients [8].

The PNI score, a nutritional biomarker, is associated with many malignancies. Qiu et al. reported that a low PNI score was associated with poor survival and disease-free survival in rectal cancer patients. As serum albumin was specifically used in the calculation of PNI, it was found to be lower in gastrointestinal malignancies. Although our study was performed in PTC patients, and we did not find very low serum albumin levels, we found similar results to Qiu et al. We may attribute the statistically significant results to the fact that PNI is a reliable biomarker [9].

In the literature, there are studies on the HALP score and its prognostic aspects in colorectal cancers, esophageal carcinomas, hepatocellular carcinomas, pancreatic carcinomas, renal cell carcinomas, and head and neck carcinomas.

In hepatocellular carcinoma, pancreatic carcinoma, and esophageal carcinoma, a low HALP score was associated with low overall survival and poor prognosis [10-12].

Since PTC has very favorable results in terms of overall survival, patients were not compared in terms of overall survival. When the current literature is evaluated, we can predict that patients with cancer-related anemia, hypoalbuminemia, and low platelet levels are patients with low expectations in terms of survival [13]. PTC is positively differentiated from all other malignancies with 10, 15, and 20-year survival rates of 97%, 95%, and 90%, respectively [14]. Therefore, it was not appropriate to evaluate the survival status of the patients with any parameter including the HALP score.

Similarly, studies on HALP score in breast cancer have been conducted to predict axillary lymph node metastasis, not survival. In a study by Duran et al., a low HALP score was associated with aggressive tumor activity such as advanced tumor and axillary lymph node positivity, but they suggested that the HALP score should not be used alone to predict axillary lymph node metastasis [2]. In correlation with our results, since PTC and breast cancer are cancer types that do not have much effect on immunonutritional parameters, we can say that the predictive power of lymph node metastasis is not like other malignancies.

We did not find a significant effect of the HALP score on lymph node metastasis in PTC as in other malignancies. This is the first study to investigate the relationship between the PTC and HALP scores. Lymph node metastasis status is the most important and controversial issue affecting the prognosis and management of PTC. Prophylactic central lymph node dissection and the extent of surgery in clinically node-negative patients is still a controversial issue with different opinions [15]. Therefore, although various methods have been tried to predict preoperative lymph node metastasis, we could not obtain a significant result in our study.

Vascular invasion, lymphatic invasion, and extra-thyroidal invasion are risk factors for lymph node metastasis [16,17]. However, in our study, no significant result was found with the HALP score in terms of vascular invasion, lymphatic invasion, and extra-thyroidal spread of the tumor in addition to lymph node metastasis.

It is clear that hepatocellular carcinoma, pancreatic carcinoma, and esophageal carcinoma are malignancies that cause hypoalbuminemia, anemia, and low platelet levels in patients. This finding may be due to the fact that PTC generally does not affect blood biochemical parameters as much as other malignancies [18].

We can say that the limitation of our study is its retrospective design. Many biomarkers were examined, but only PNI was found to be statistically significant. We believe that this should be supported prospectively in series with a larger number of patients.

## Conclusions

Nowadays, low-cost non-invasive tests and various scoring methods are being used in order to predict both the diagnosis and prognosis of many diseases. The HALP score is one of these scoring methods and some studies have shown its clinical efficacy in some diseases. Since there is no previous study in the literature on the HALP score and PTC, our study has the value of being the first. It should be noted that the PNI score can be used in many malignancies in the preoperative period and can be calculated and relied upon for central regional lymph node metastasis in PTC patients.

We think that non-invasive predictive tests are an open subject for further research.
